# 241. Early switch from intravenous to oral antibiotics in patients with Gram-negative bacteremia – a target trial emulation

**DOI:** 10.1093/ofid/ofad500.314

**Published:** 2023-11-27

**Authors:** Sandra Tingsgård, Simone Bastrup Israelsen, Christian Østergaard, Thomas Benfield

**Affiliations:** Copenhagen University Hospital - Hvidovre, Hvidovre, Hovedstaden, Denmark; Copenhagen University Hospital - Hvidovre, Hvidovre, Hovedstaden, Denmark; Copenhagen University Hospital - Hvidovre, Hvidovre, Hovedstaden, Denmark; Copenhagen University Hospital, Hvidovre, Hovedstaden, Denmark

## Abstract

**Background:**

Gram-negative bacteremia is a global health concern, that continues to cause significant morbidity and mortality. Intravenous (IV) antibiotics are typically the first-line treatment; however, it is unclear when it is appropriate to switch to oral antibiotics in order to maintain positive outcomes. An early switch from intravenous to oral antibiotics may have several advantages, including reduced hospital stay, lower healthcare costs, and improved patient quality of life. This study investigates the effectiveness of early switch to oral antibiotics in patients with Gram-negative bacteremia using a target trial framework.

**Methods:**

We outlined the protocol for a target trial to estimate the effect of 1) switching to oral antibiotics within four days after initial blood culture compared with 2) continuing IV antibiotic treatment throughout the treatment course on 90-day all-cause mortality. We then emulated the trial using a cohort of adult patients with Gram-negative bacteremia from 2018 through 2022 admitted to four hospitals in the Capital Region of Denmark. Patients who were immunosuppressed, transitioned to hospice care, had polymicrobial infection, were not clinically stable within four days of blood culture, or did not receive adequate empirical antibiotic treatment were excluded. Inverse probability of treatment weighting was applied to adjust for confounding. Intention-to-treat analysis was performed using pooled logistic regression to estimate absolute risk, risk difference, and risk ratio. 95% confidence intervals (CI) were computed using bootstrapping.

**Results:**

1088 patients were eligible for inclusion in the study. Of these, 582 patients transitioned early to oral antibiotic treatment and 506 patients continued IV treatment. The absolute risk of 90-day all-cause mortality was 11.6% (95% CI 9.4;13.7) for patients switching early to oral antibiotics, and 13.4% (95% CI 11.2; 15.5) for patients continuing IV. The risk difference was -1.8% (95% CI -4.9; 1.3) and the risk ratio was 0.99 (95% CI 0.67; 1.13).

**Figure:**
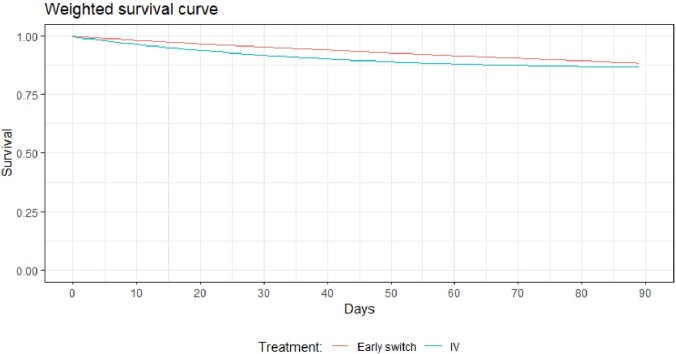

**Conclusion:**

Our findings indicate that an early switch to oral antibiotic treatment within four days may be a safe and effective alternative to continued IV treatment in patients hospitalised with uncomplicated Gram-negative bacteremia.

**Disclosures:**

**All Authors**: No reported disclosures

